# Clinical impact of quality nursing combined with health education pathway on quality of life and sleep in severe aplastic anemia patients complicated with infection: a randomized clinical trial

**DOI:** 10.3389/fmed.2025.1607485

**Published:** 2025-08-06

**Authors:** Wei Shen, Feng Wei

**Affiliations:** Department of Hematology, The First Affiliated Hospital of Soochow University, Suzhou, China

**Keywords:** health education, quality nursing, quality of life, severe aplastic anemia, sleep

## Abstract

**Objective:**

This research aimed to elucidate the clinical impact of quality nursing combined with a health education pathway on the quality of life and sleep in patients with severe aplastic anemia (SAA) complicated by infection.

**Methods:**

A total of 130 SAA patients complicated with infection, admitted to the Hematology Department of our hospital between February 2022 and February 2024, were selected as research subjects. Using the number table method, participants were randomly divided into an observation group (OG) and a control group (CG), with 65 patients in each group. Patients in the CG received conventional nursing care. In addition to conventional nursing care, those in the OG received quality nursing care combined with health education. The health knowledge awareness rate, quality of sleep, quality of life, inflammatory indicators, immune function indicators, improvement of clinical symptoms, and occurrence of adverse reactions were analyzed and compared.

**Results:**

After the intervention, the health knowledge awareness rate was significantly higher among patients in the OG than those in the CG (*p* = 0.002). The Pittsburgh Sleep Quality Index (PSQI) scores in the OG were significantly lower, while sleep duration was significantly longer compared to the CG (*p* < 0.0001). Additionally, quality-of-life scores in the OG were significantly higher than those in the CG (*p* < 0.0001). Patients’ PSQI scores were inversely proportional to their quality-of-life scores in both groups (*p* = 0.0044, *p* = 0.0007, *p* = 0.0003, and *p* < 0.0001). Conversely, sleep duration was positively correlated with quality-of-life scores in both groups (*p* = 0.005, *p* < 0.0001, *p* = 0.0005, and *p* < 0.0001). After the intervention, levels of C-reactive protein (CRP), procalcitonin (PCT), and interleukin-6 (IL-6) were significantly lower in the OG than in the CG, while the CD4/CD8 ratio in the OG was significantly higher than that in the CG (*p* < 0.0001). Additionally, the time to infection control, time for body temperature to return to normal, time for cough disappearance, and time for lung rales disappearance were all significantly shorter in the OG than in the CG (*p* < 0.0001). The total incidence of adverse reactions in the OG depicted a reduction in comparison to that in the CG (*p* = 0.013).

**Conclusion:**

The combined application of quality nursing and a health education pathway in nursing practices of SAA patients demonstrates significant clinical benefits. It can improve patients’ health knowledge awareness, enhance sleep quality and overall quality of life, enhance immune function, attenuate adverse reactions, and facilitate recovery. This approach is worth promoting further in clinical practice.

## 1 Introduction

Bone marrow failure syndrome is a group of diseases characterized by insufficient blood cell production (1). Aplastic anemia (AA) is one of the typical types of bone marrow failure syndrome, presenting with a decrease in all blood cells along with reduced bone marrow proliferation (2). AA patients may experience symptoms such as pale complexion, palpitations, bleeding, and physical weakness, which can easily lead to related complications such as anemia, infection, and hemochromatosis (a result of red blood cell transfusion) (3, 4). In addition, patients with AA have significantly lower immunity than ordinary people. When they contract SARS-CoV-2 or other respiratory diseases, they will face multiple burdens, such as the deterioration of underlying conditions, an increased risk of complications, and greater difficulty in treatment (5). Paroxysmal nocturnal hemoglobinuria (PNH) is a rare disease that affects the hematopoietic stem cells and causes bone marrow failure (6, 7), and the main manifestations include paroxysmal nocturnal hemoglobinuria, anemia, thrombosis, and infection. AA or myelodysplastic syndrome (MDS) is related to the syndrome of bone marrow failure and is a subtype of PNH (8, 9). MDS is a highly heterogeneous myeloid clone of hematopoietic stem cells, mainly caused by abnormal myeloid cell development, resulting in ineffective hematopoiesis and refractory blood cell deficiency (10). Due to the connections and differences among PNH, AA, and MDS, the distinction in diagnosis and treatment of these three diseases is of great significance.

Severe aplastic anemia (SAA) has an acute onset with progressive worsening of anemia and is often accompanied by severe infections or bleeding tendencies. It poses a serious threat to patients’ lives and involves expensive treatment, placing a significant economic burden on both their families and society (11, 12). Currently, immune suppression, hematopoietic growth factors, and targeted antibiotic treatments are major treatments for SAA, which can effectively control the condition, enhance hematopoietic function, and help improve patients’ prognosis (13, 14). Moreover, severe and systemic infections can develop in SAA patients, increasing the morbidity that these patients experience. Due to severe neutropenia, inflammation cannot be localized, and in severe cases, this can lead to sepsis, worsening bleeding, endangering patients’ lives, and elevating the mortality rate (15). Thus, for SAA patients, it is necessary to implement comprehensive nursing interventions to effectively control patients’ infections and enhance the treatment effect after coexisting with infection. Quality nursing refers not only to the strengthening of basic content but also to the deepening of nursing connotation, with the aim of improving the level of nursing services (16, 17). It can protect the life safety of patients, improve their sleep quality, quality of life, and other aspects, facilitate patients to accelerate their physical recovery, and elevate treatment efficacy with a more positive and stable mentality. Health education is crucial in nursing practices and is a vital measure to enhance patients’ health knowledge awareness, which can markedly elevate patients’ treatment enthusiasm and cooperation, enhance self-care effectiveness, and improve clinical treatment outcomes and prognosis (18). However, at present, there are no reports on the impact of the combination of quality nursing and health education pathway on clinical outcomes in patients with SAA complicated by concurrent infections.

Therefore, this study aimed to elucidate the clinical impact of quality nursing combined with the health education pathway on the quality of life and sleep quality of SAA patients complicated by infection. Our study demonstrated that, in the nursing of SAA patients, integrating quality nursing with the health education pathway achieved remarkable clinical effects, which can enhance patients’ health knowledge, improve their sleep and quality of life, strengthen their immune function, and alleviate adverse reactions. Our study may provide advisory guidance for the nursing practices of SAA patients.

## 2 Materials and methods

### 2.1 General data

A total of 130 SAA patients complicated by infection, admitted to the Hematology Department of our hospital from February 2022 to February 2024, were selected and randomly divided into an observation group (OG) and a control group (CG) using a random number table method, with 65 cases each. This research was approved by the Medical Ethics Committee of our hospital (Approval Number: SZ2022-L004). Informed consent was obtained from all patients.

### 2.2 Sample size calculation

In this study, we conducted a power analysis using G*Power 3.1.9.7 software to determine the sample size required to detect statistical differences. With an *α* value of 0.05 and a 90% power analysis, the study results indicated that 65 patients were needed in each group. Therefore, to draw reliable conclusions, the sample size of the study was set at 65 patients per group.

### 2.3 Randomization and blinding

A group randomization design was adopted for random grouping. The random allocation sequence was generated by a computer. The confidentiality of the allocation was achieved through continuous numbering, sealing, and opaque envelopes. After meeting the inclusion criteria, patients were randomly assigned to CG or OG at a 1:1 ratio. This study was a single-blind study, and the participants were unaware of their allocation.

### 2.4 Nursing measures

The CG received conventional nursing care, which included the following:

1. A brief introduction to relevant disease-related knowledge, monitoring of patients’ physical conditions, and prompt treatment when signs of bleeding or infection were observed.
2. The nurses encouraged patients to adopt healthy living habits and assisted them in engaging in appropriate physical exercises.

Based on conventional nursing, the OG received quality nursing + health education nursing. The specific content was as follows:

1. A quality nursing team was established to carry out nursing measures. Before the start of the nursing practices, a centralized training program was conducted for the team members with the aim of enhancing the service awareness and nursing skills of the nurses and cultivating their professional qualities in providing high-quality nursing. The nurses underwent an assessment 1 day before the training concluded. Only those who passed the assessment could officially become members of the team. The team members maintained good communication, actively learned about the patient’s condition and basic information, analyzed the nursing risks based on previous nursing experience, conducted a comprehensive assessment, and ultimately determined the plan.
2. Health education. The nurses provided health education to patients and communicated with them often to gain a deep understanding of their daily habits, identifying any unhealthy behaviors of patients. The nurses informed patients of the positive impact of healthy habits on improvement and urged them to correct their bad behaviors.
3. Strengthening basic nursing. The nurses had a comprehensive understanding of the actual needs of patients, tried their best to meet those needs, and guided patients to take bed rest after confirming the condition of the disease. The nurses set up barriers beside the beds to prevent patients from falling off the beds and frequently monitored the patients’ vital signs. For patients showing signs of infection, the nurses followed the doctor’s instructions to administer anti-infection treatment to the patients. Before administering the medication, the nurses verified the patient’s basic information, explained the usage method of the medication and possible adverse reactions to the patient, introduced methods for identifying adverse reactions to the patient, and promptly contacted the doctor if the patient experienced any discomfort. The patient’s medication dosage was reduced or stopped in accordance with the doctor’s instructions. The nurses regularly carried out nursing tasks such as regular cleaning, ventilation, and disinfection to ensure that the temperature, humidity, and cleanliness of the ward environment meet the requirements, creating a safe and comfortable ward environment for patients. For patients suffering from insomnia and facing difficulty in falling asleep, according to the doctor’s advice, the nurses instructed the patients to take the prescribed medications to improve their sleep quality.
4. Prevention of complications. The nurses distributed educational brochures to the patients, in which the complications of aplastic anemia are explained in simple language to the patients, particularly the knowledge about infections and bleeding symptoms. The nurse played health promotion videos in the ward at an appropriate time to further enhance patients’ understanding of the disease and its common complications.
5. Daily life guidance. The nurses enhanced the monitoring of patients’ body temperature, paid attention to the patients’ coughing and pain during urination, and ensured proper skin cleaning for the patients. The nurses explained the isolation and protection procedures in detail to the patients and their families and guided them in preparing the necessary items. Patients who were infected underwent sitz baths with potassium permanganate solution to prevent perianal necrotizing ulcers. When providing sitz bath instructions in the isolation ward, the nurses wore sterile clothing appropriately. The nurses informed the patients to consume light and easily digestible food, avoid foods with spines, bones, or that are too hot, drink plenty of water, and ensure smooth defecation.

### 2.5 Observation indicators

1. Health knowledge awareness rate: The nurses used the hospital’s self-made health knowledge forms to determine the scope of health knowledge inquiries and randomly asked the patients 10 questions. The test content covers four aspects: the causes of the disease, clinical manifestations, treatment plans, and nursing measures. If the patients could answer six or more questions, it indicated that they had some understanding; otherwise, it indicated that they did not have such an understanding. The formula for the health knowledge awareness rate is as follows: health knowledge awareness rate = the number of cases who knew health knowledge/the total number of cases.
2. Quality of sleep: After intervention (2 weeks after intervention), the quality of sleep between both groups was compared. The sleep quality of patients was evaluated with the Pittsburgh Sleep Quality Index (PSQI) (19). PSQI is a commonly applied tool to evaluate the sleep quality of patients, with a total score of 21 points. The scores have a negative correlation with patients’ sleep quality, with higher scores indicating poorer sleep quality.
3. Quality of life: After intervention (2 weeks after intervention), the postoperative quality of life between both groups was compared. The postoperative quality of life of patients was comprehensively evaluated with the Short-Form 36 (SF-36) scale (20). This scale includes multiple dimensions, such as role-physical health, mental health, bodily pain, and vitality, with a scoring range of 0–100 points for each dimension. The scores have a positive correlation with patients’ quality of life, with higher scores indicating a better quality of life.
4. Inflammatory indicators and immune function indicators. After intervention (2 weeks after intervention), 5 mL of peripheral venous blood was drawn from patients on an empty stomach under sterile conditions and centrifuged at a speed of 3,000 r/min for 5 min. The inflammatory markers, such as serum C-reactive protein (CRP) and procalcitonin (PCT) levels, were detected through the Beckman Coulter AU5800 fully automated biochemical analyzer. The inflammatory markers, such as interleukin-6 (IL-6), were detected with the enzyme-linked immunosorbent assay (ELISA). The immune function indicators [cluster of differentiation 4 positive (CD4^+^), cluster of differentiation eight positive (CD8^+^)] were detected through Beckman Coulter CytoFLEX S flow cytometry, and the CD4/CD8 ratio was calculated.
5. Improvement of clinical symptoms and occurrence of adverse reactions: The time for infection control, body temperature to return to normal, cough disappearance, and lung rales disappearance in both groups was recorded. The number of adverse reactions, such as acne, liver damage, and hand and foot tremors, during the treatment process was recorded, and the total incidence of adverse reactions was calculated.

### 2.6 Statistical analysis

SPSS 27.0 statistical software was used for data analysis. The measurement data conforming to a normal distribution were represented by mean ± standard deviation (*x* ± *s*), followed by a t-test for intergroup comparison. The Shapiro–Wilk test was used to ensure the normal distribution before the t-test was conducted. The counting data were expressed as a percentage (%), followed by a chi-squared test for intergroup comparison. The correlation of quality of sleep with quality of life was analyzed using Pearson’s correlation analysis. The difference was statistically significant (*p* < 0.05).

## 3 Results

### 3.1 General data of patients between the two groups

The CG comprised 40 men and 25 women, and the mean age was (53.76 ± 4.17) years. There were 24 cases of lung infection, 25 cases of oral infection, and 16 cases of nasal infection. The OG included 37 men and 28 women, and the mean age was (53.60 ± 4.11) years. There were 25 cases of lung infection, 26 cases of oral infection, and 14 cases of nasal infection. There were no significant differences in gender, age, and infection site between the two groups (*p* = 0.592, *p* = 0.826, and *p* = 0.917; Table 1), indicating comparability.

**Table 1:** General data of patients between the two groups [*n* (%)/(*x* ± *s*)].

Variable	CG (*n* = 65)	OG (*n* = 65)	*p* value
Sex			0.592
Male	40 (61.54)	37 (56.92)	
Female	25 (38.46)	28 (43.08)	
Age (years)	53.76 ± 4.17	53.60 ± 4.11	0.826
Infection site			0.917
Lung infection	24 (36.92)	25 (38.46)	
Oral infection	25 (38.46)	26 (40.00)	
Nasal infection	16 (24.62)	14 (21.54)	

CG, control group; OG, observation group.

### 3.2 Quality nursing combined with a health education pathway elevates the health knowledge awareness rate in SAA patients complicated by infection

After intervention, the health knowledge awareness rate in the OG was significantly higher than that in the CG (*p* = 0.002; Table 2).

**Table 2:** Impact of joint intervention on health knowledge awareness rate [*n* (%)].

Variable	CG (*n* = 65)	OG (*n* = 65)	*p* value
Number of health knowledge awareness cases	48	61	0.002
Health knowledge awareness rate	73.85	93.85	

CG, control group; OG, observation group.

### 3.3 Quality nursing combined with a health education pathway improves the quality of sleep in SAA patients complicated by infection

**Figure 1:**
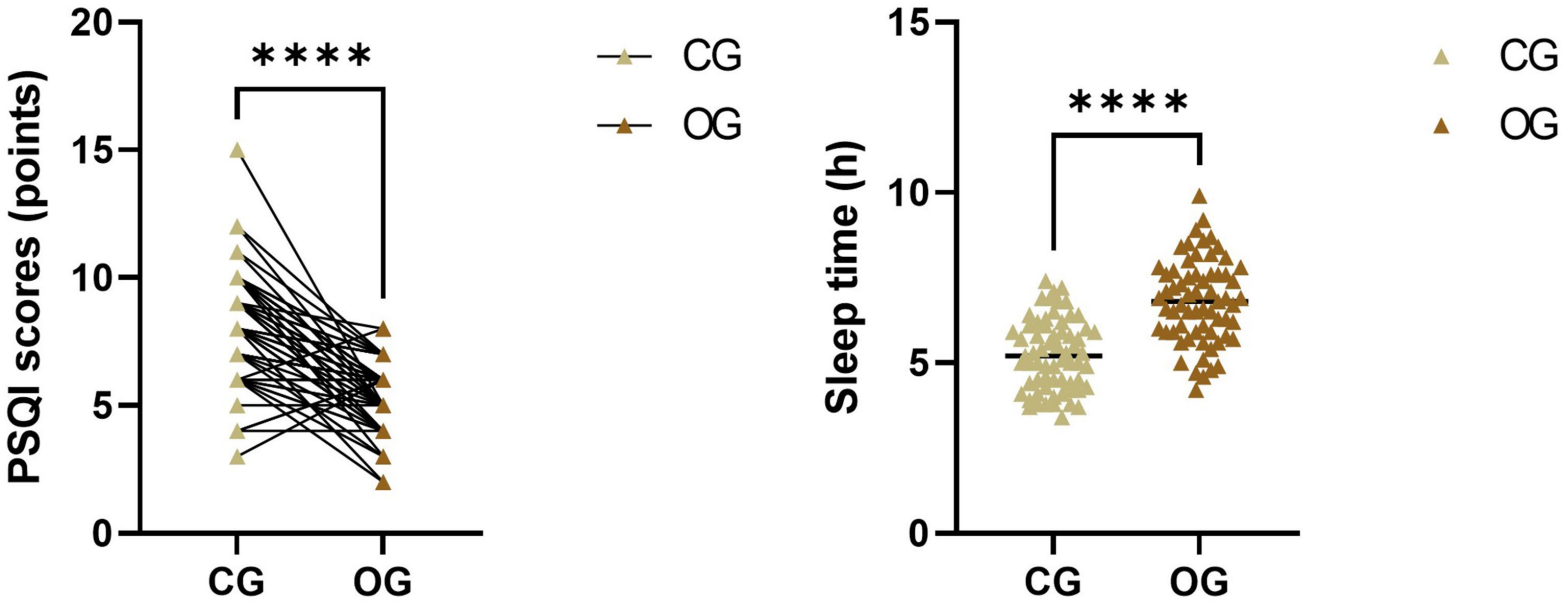
Impact of joint intervention on quality of sleep. ****p < 0.0001. CG, control group; OG, observation group; PSQI, Pittsburgh Sleep Quality Index.

After intervention, PSQI scores in the OG showed a significant reduction compared to the CG, while sleep time in the OG increased compared to the CG (*p* < 0.0001; Figure 1).

### 3.4 Quality nursing combined with a health education pathway enhances the quality of life in SAA patients complicated by infection

**Figure 2:**
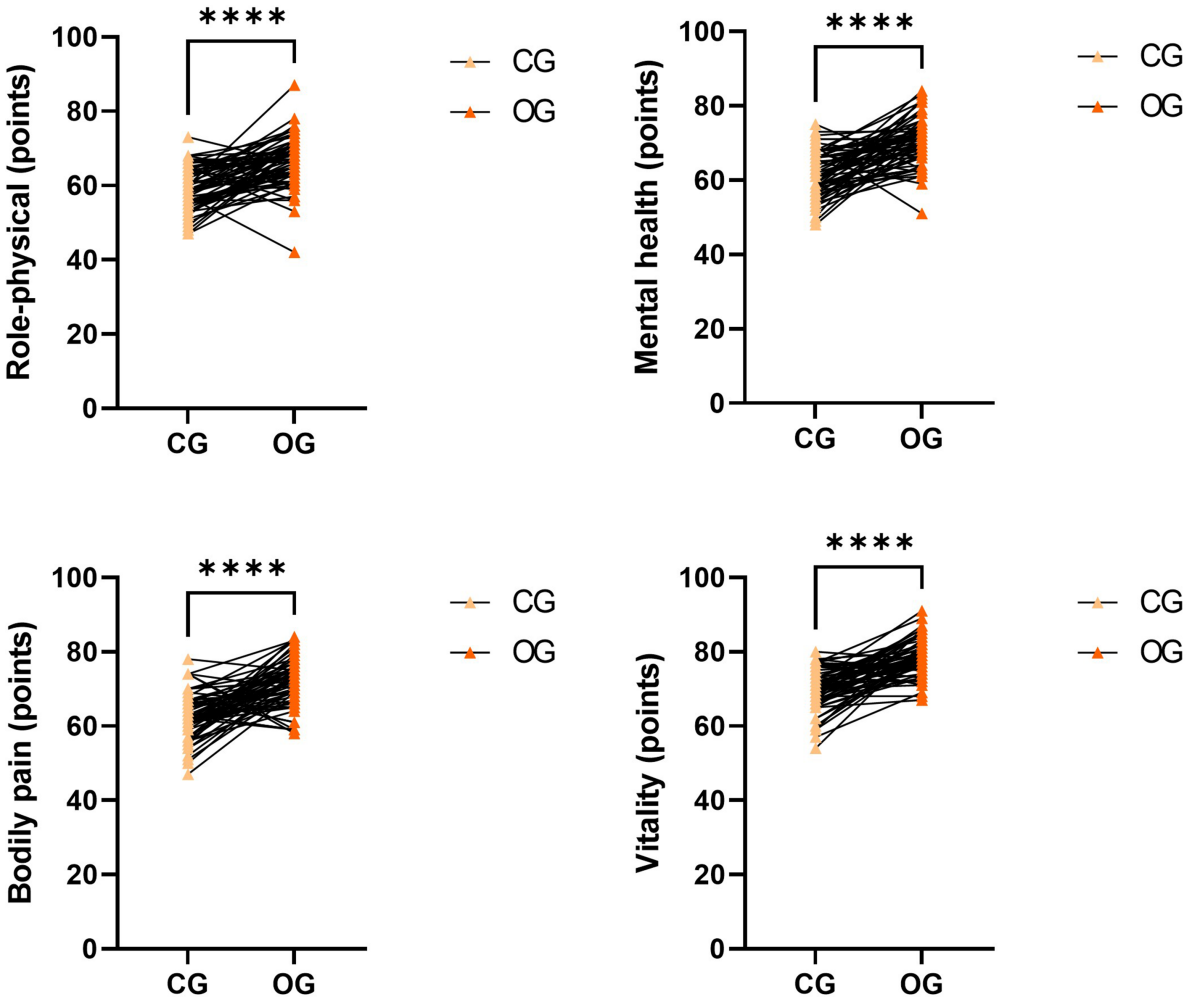
Impact of joint intervention on quality of life. ****p < 0.0001. CG, control group; OG, observation group.

After intervention, quality-of-life scores in the OG showed a significant increase compared to those in the CG (*p* < 0.0001; Figure 2).

### 3.5 The elevation of quality of sleep has a close relation to the improvement of quality of life in patients receiving quality nursing combined with a health education pathway

**Figure 3:**
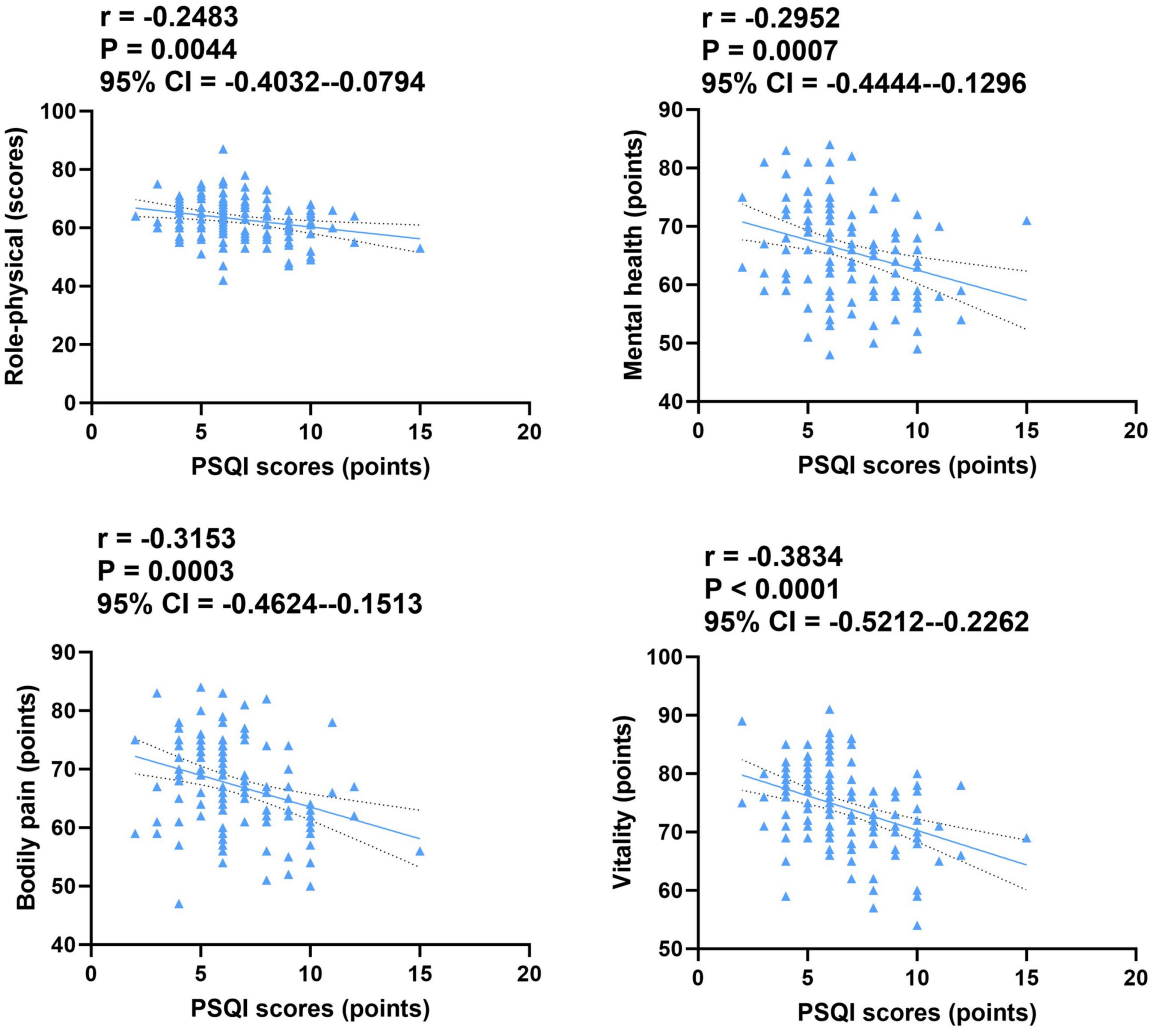
Correlation of PSQI scores with quality of life in both groups. CI, confidence interval; PSQI, Pittsburgh Sleep Quality Index.

**Figure 4:**
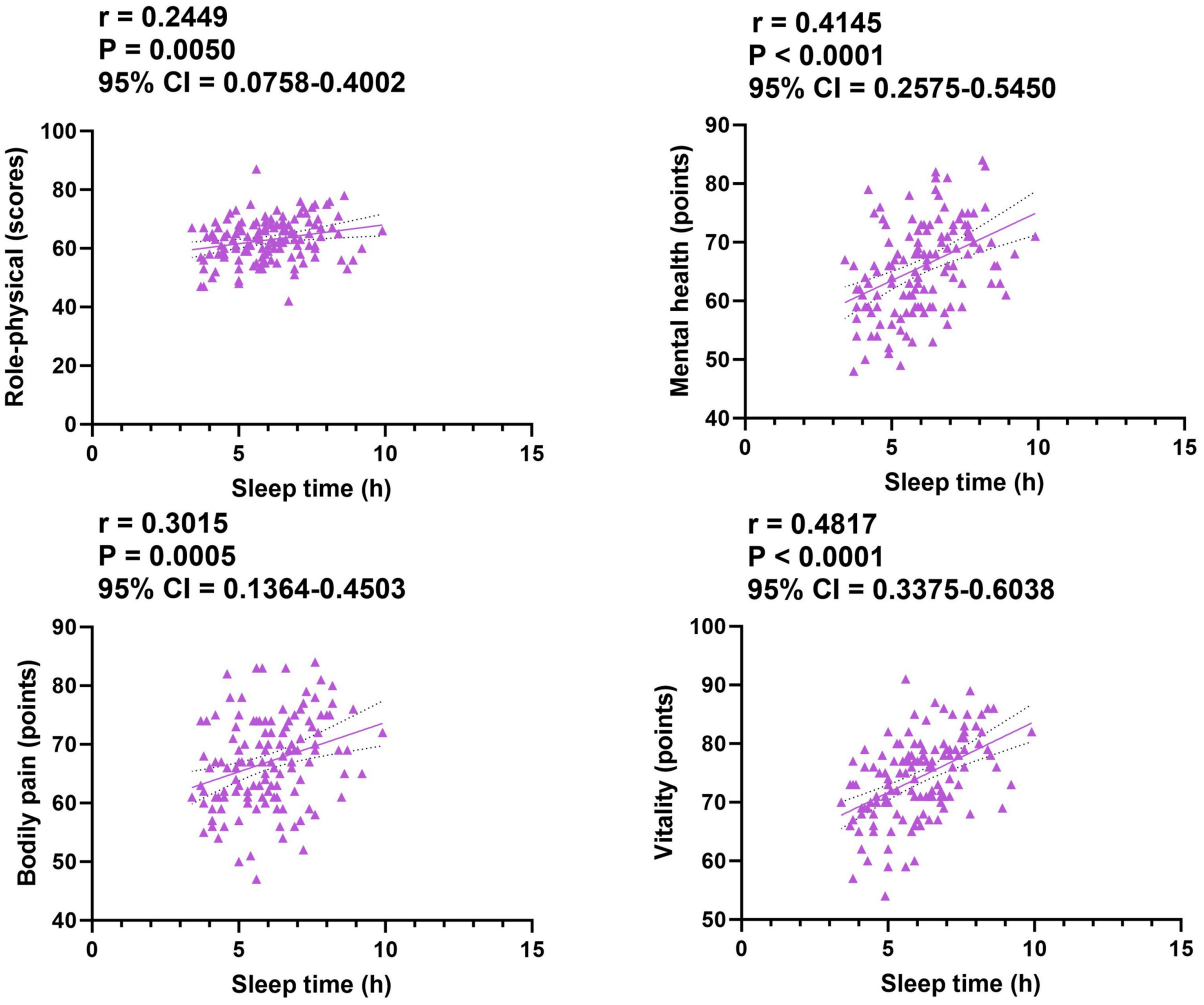
Correlation of sleep time with quality of life in both groups. CI, confidence interval.

The patients’ PSQI scores were inversely correlated with their quality-of-life scores in both groups (*p* = 0.0044, *p* = 0.0007, *p* = 0.0003, and *p* < 0.0001; Figure 3). Conversely, patients’ sleep time was positively correlated with their quality-of-life scores in both groups (*p* = 0.0050, *p* < 0.0001, *p* = 0.0005, and *p* < 0.0001; Figure 4).

### 3.6 Quality nursing combined with a health education pathway mitigates inflammation and improves immune function in SAA patients complicated by infection

**Figure 5:**
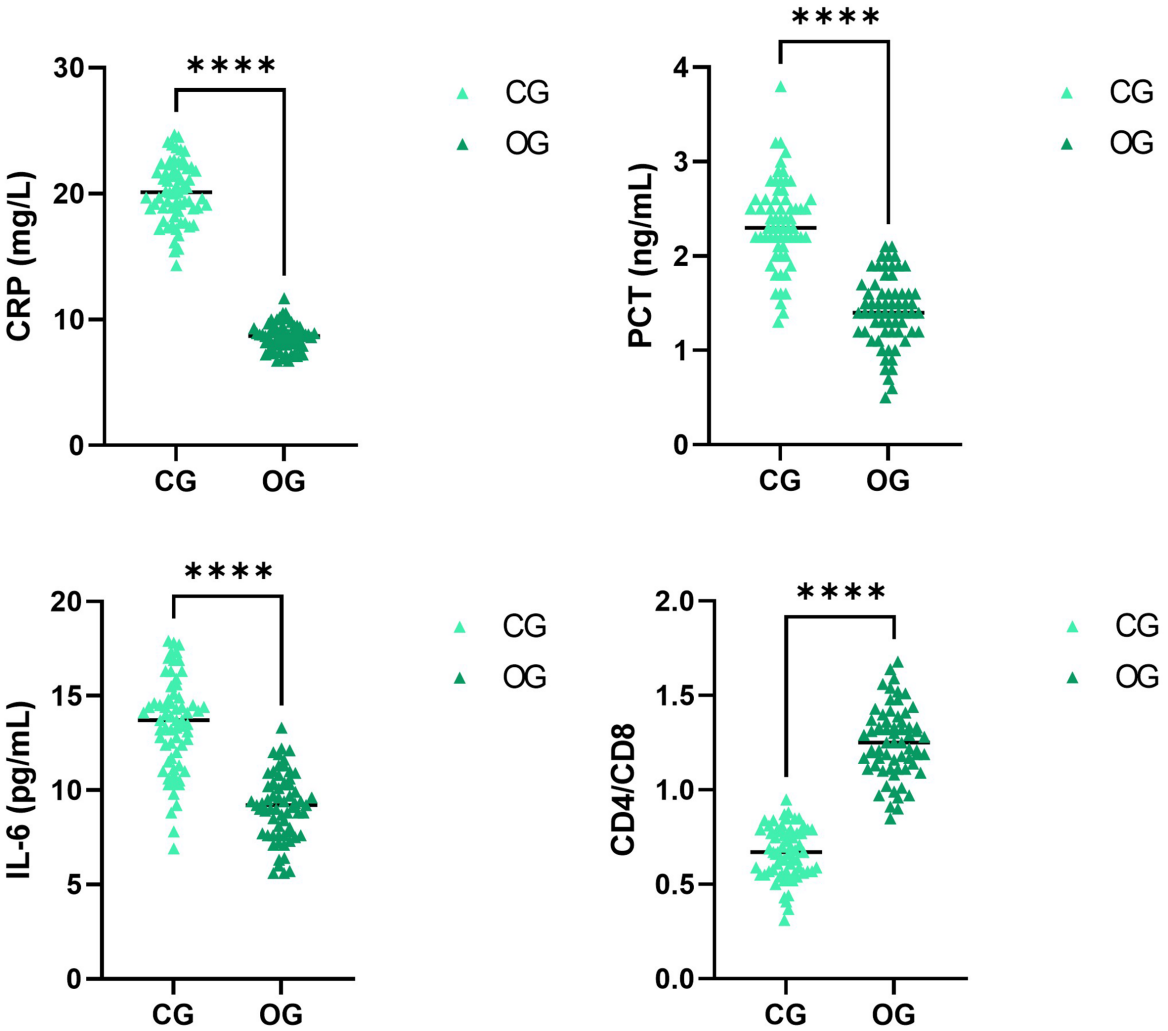
Impact of joint intervention on inflammation and immune function. ****p < 0.0001. CG, Control group; CD4/CD8, Cluster of differentiation four positive/cluster of differentiation eight positive; CRP, C-reactive protein; IL-6, interleukin-6; OG, observation group; PCT, procalcitonin.

After intervention, CRP, PCT, and IL-6 levels in the OG were significantly lower than those in the CG, while the CD4/CD8 ratio in the OG was significantly higher compared to the CG (*p* < 0.0001; Figure 5).

### 3.7 Quality nursing combined with a health education pathway ameliorates clinical manifestations and alleviates the incidence of adverse reactions in SAA patients complicated by infection

**Figure 6:**
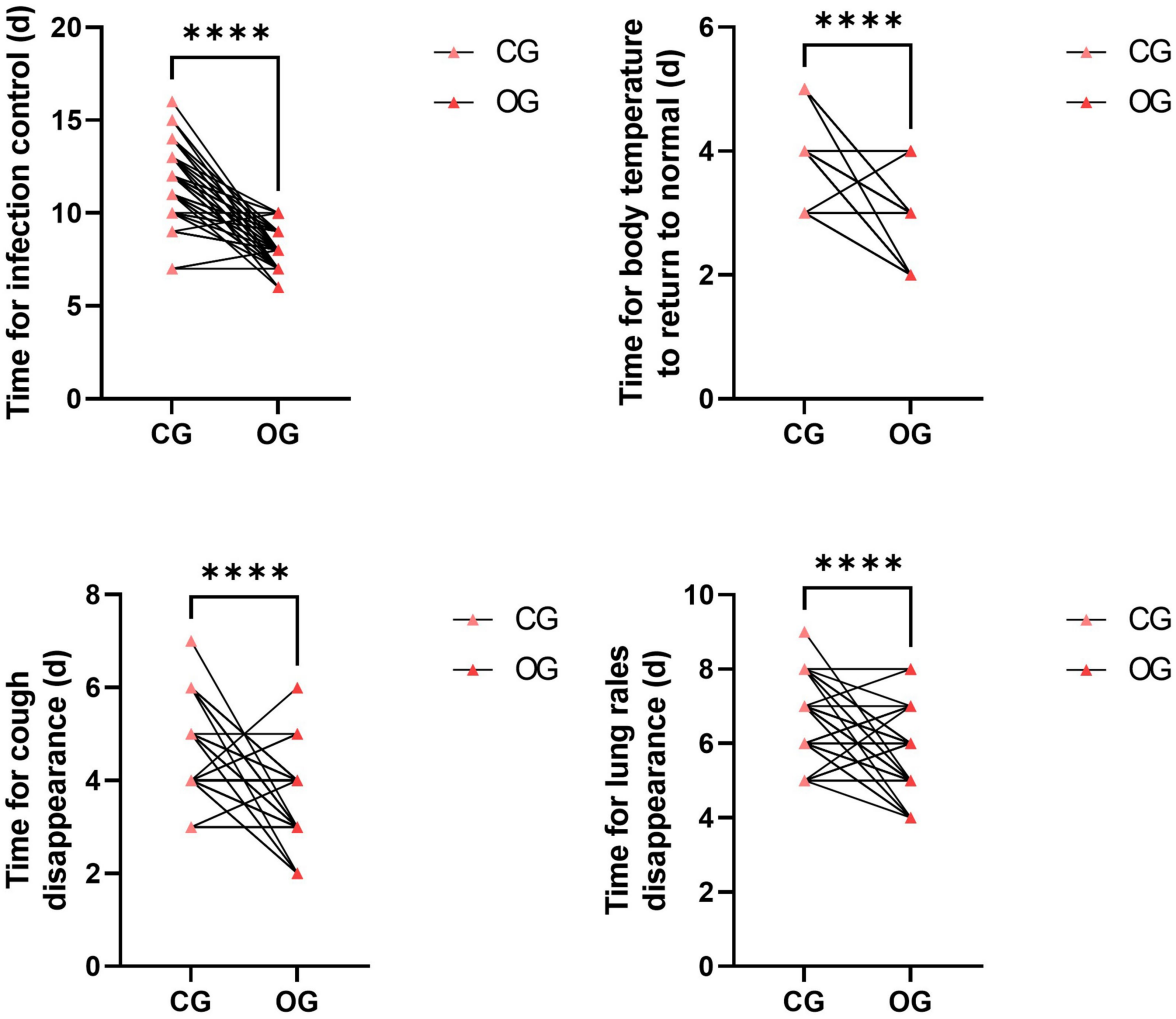
Impact of joint intervention on clinical manifestations. ****p < 0.0001. CG, control group; OG, observation group.

After intervention, the time required for infection control, body temperature to return to normal, cough disappearance, and lung rales disappearance were all significantly shorter in the OG compared to the CG (*p* < 0.0001; Figure 6). Additionally, the total incidence of adverse reactions in the OG was significantly lower than the CG (*p* = 0.013; Table 3).

**Table 3:** Impact of joint intervention on adverse reactions [n (%)].

Variable	CG (*n* = 65)	OG (*n* = 65)	*p*-value
Acne	4 (6.15)	1 (1.54)	0.013
Liver damage	5 (7.69)	2 (3.08)	
Hand and foot tremors	3 (4.62)	0 (0.00)	
Total incidence of adverse reactions	12 (18.46)	3 (4.62)	

CG, control group; OG, observation group.

## 4 Discussion

Our research conducted a randomized clinical trial aimed at exploring the clinical effects of a quality nursing approach combined with health education on patients with ASS who have infections. The main findings revealed that, in the nursing practices for SAA patients, integrating quality nursing with the health education pathway could enhance patients’ health knowledge level, improve their sleep and quality of life, strengthen their immune function, and alleviate adverse reactions, which is worthy of further promotion in clinical practice.

SAA is a group of diseases caused by impaired hematopoietic function in the bone marrow. The symptoms of SAA are quite severe and may lead to repeated episodes of the disease, which can easily cause complications such as bleeding or infection, thereby reducing the patient’s quality of life and increasing their financial burden (21). Multiple patients may experience negative psychological reactions and gradually refuse treatment, reducing overall clinical efficacy (22). Thus, improving the quality of clinical nursing practices is crucial for the recovery of SAA patients.

When understanding the concept of quality nursing, one can approach it from three perspectives: patient satisfaction, social satisfaction, and government satisfaction. Compared with basic nursing services, the quality nursing model has undergone changes. The implementation of a responsibility system has achieved a significant leap in “nursing practices quality,” establishing a better image for nurses. In addition, based on the cooperation of the nursing team, more time can be left for the patients, truly placing them at the core of the treatment. Meanwhile, nurses are encouraged to actively acquire professional knowledge and actively participate in clinical nursing practices. They provide patients with comprehensive, systematic, and scientific health information, aiming to meet their health knowledge needs as much as possible, cultivate patients’ self-care awareness, enhance patients’ self-care ability, thereby effectively controlling the progression of patients’ diseases, preventing the occurrence of adverse complications, and ensuring the quality of patients’ lives (23, 24). In addition, health education plays a vital role in clinical nursing. It can help patients enhance their cognitive abilities and relieve stress, enabling them to actively seek medical assistance. This is a key guarantee for improving clinical efficacy (25).

Herein, the results of our study indicated that, compared with the CG, the OG had a higher awareness rate of health knowledge, better sleep quality, and higher quality of life, suggesting that the quality nursing approach combined with health education could promote the health knowledge level, improve the sleep quality, and promote the quality of life of SAA patients. The reasons were as follows: (1) through training and assessment, qualified nurses were selected to establish a high-quality nursing team. These nurses actively studied relevant theoretical knowledge, which provided favorable conditions for formulating personalized care measures for patients; (2) nurses carried out health education work based on the health information model and the individual differences of patients, making the nursing practices have clear purposes and plans, significantly improving the work effectiveness; and (3) the application of health education enabled patients to have a detailed understanding of their diseases, reduced patients’ psychological pressure, alleviated patients’ negative emotions, and thus patients could cooperate actively with the treatment. Consistently, Wen and Liu proposed that health education combined with cluster-based nursing could promote healthy behavior and help improve the quality of life in patients with gestational hypertension (26). Fang et al. suggested that glioma patients were given high-quality care during the course of concurrent radiotherapy and chemotherapy, which reduced the bad mood of the patient, reduced the stress response of the patient, and improved the quality of sleep and the quality of life of the patient (27).

Immune activation is one of the crucial pathogenic mechanisms in SAA-infected patients, and the CD4/CD8 ratio in the bone marrow of patients is markedly elevated relative to that of normal individuals (28). CRP, PCT, and IL-6 are common diagnostic markers for bacterial infections, and when inflammation occurs, CRP, PCT, and IL-6 are released in large quantities and can be applied to evaluate treatment efficacy in patients (29). Herein, our study manifested that, compared with the CG, the OG had lower levels of CRP, PCT, and IL-6 and a higher CD4/CD8 ratio. This suggested quality nursing approach, combined with health education, could effectively control infections and enhance the immune function of SAA patients. The reason was that, in the model combining quality nursing with health education, nurses could effectively control patients’ conditions by implementing various treatment and nursing measures. Moreover, nurses effectively enhanced patients’ immune functions through targeted daily life guidance. Consistent with our findings, it has been reported that high-quality nursing can decrease inflammation and improve prognosis for postoperative patients with advanced non-small cell lung cancer (30).

Furthermore, our study indicated that compared to the CG, the OG had shorter times for infection control, shorter times for body temperature to return to normal, shorter times for cough disappearance, shorter times for lung rales disappearance, and a lower incidence of adverse reactions. All of these results implied that a quality nursing approach combined with health education could effectively promote the recovery of SAA patients. The reasons were as follows: (1) the quality nursing model ensured the effective implementation of nursing intervention measures through reasonable division of labor; (2) health education enhanced patients’ effective understanding of the disease and improved their medication compliance; and (3) the combined nursing model focused on observing patients’ vital signs and selecting appropriate and safe medications for treatment, which effectively ensured the treatment outcome, reduced the incidence of adverse reactions in patients, and contributed to their recovery. Similarly, it has been documented that high-quality nursing intervention based on humanistic care combined with the project teaching method for acute leukemia patients undergoing chemotherapy can effectively relieve negative emotions, improve clinical nursing satisfaction, and reduce adverse reactions during chemotherapy (31).

Our research has some limitations. First, our sample size is relatively small, which may lead to deviations between the data results and actual values. Second, our research was a single-center study, and the sample was not representative, which may not accurately reflect the characteristics of a broader population. Third, our study did not discuss possible confounding factors (such as the severity of the disease and the effect of the drugs), which may have led to our results being less accurate and comprehensive. Fourth, our research did not conduct long-term follow-up observations. The effect of quality nursing combined with health education pathways on the long-term prognosis of SAA patients is currently unclear. Therefore, more multi-center, large-scale, and long-term studies should be conducted in the future to further verify our findings.

In conclusion, the combined application of quality nursing and health education pathways in the nursing of SAA patients can enhance patients’ understanding of health knowledge, improve their sleep and quality of life, strengthen their immune function, reduce the incidence of adverse reactions, and promote their recovery, which is worthy of further promotion in clinical practice. In the future, our study will explore the combined application of quality nursing and health education pathways to the long-term prognosis of SAA patients.

## Data Availability

The datasets presented in this study can be found in online repositories. The names of the repository/repositories and accession number(s) can be found in the article/supplementary material.
